# *Brucella canis* outbreak management towards rehoming strategies: The Italian experience

**DOI:** 10.1371/journal.pone.0351742

**Published:** 2026-06-30

**Authors:** Fabrizio De Massis, Nausica D’Aurelio, Michela Toro, Sara Serrani, Alessandra Sferrella, Daniele Giansante, Marusca Pantalone, Fabrizia Perletta, Chiara Di Pancrazio, Marta Maggetti, Paola Ripà, Mirella Luciani, Manuela Tittarelli, Marco Violini, Flavio Sacchini

**Affiliations:** 1 Istituto Zooprofilattico Sperimentale dell’Abruzzo e del Molise “G. Caporale”, Teramo, Italy; 2 Dipartimento di Prevenzione AST Ancona, Servizio Sanità Animale, Italy; East Carolina University Brody School of Medicine, UNITED STATES OF AMERICA

## Abstract

Canine brucellosis, caused by *Brucella canis*, is a contagious disease leading to reproductive disorders in both male and female dogs. In April 2020, Italy reported its first outbreak of *B. canis* in a central breeding kennel housing over 680 toy breed dogs. Following confirmation, animal movement was restricted, and a no-kill strategy was implemented to manage the outbreak. A structured protocol, integrating castration and neutering and serological testing, was developed to identify and rehome *B. canis*-negative dogs. This study describes the procedure used to select non-infected dogs and the follow-up monitoring protocol applied post-adoption. Over 300 dogs were successfully rehomed, with laboratory investigations confirming no occurrence of late infection in the adopted animals. These results support the inclusion of rehoming strategies as a viable option in *B. canis* outbreak management.

## 1 Introduction

Brucellosis is an infectious disease caused by bacteria of the genus *Brucella*, affecting multiple species. Among domesticated animals, canine brucellosis is less frequently reported yet remains an under-researched condition. The disease also has zoonotic potential, with reported human infections typically exhibiting a milder course compared to those caused by classical *Brucella* species [1].

*B. canis* is primarily transmitted through direct contact with infected bodily fluids, and in breeding environments, it poses significant reproductive risks [2]. Clinical manifestations include infertility, embryonic death, and late-stage abortion in females, while males may develop epididymitis, orchitis, and testicular atrophy [3]. In addition to symptoms affecting the reproductive sphere, although less frequently, *B. canis* infection can cause disorders in other organs as well, such as discospondyliafricatis [4], osteomyelitis affecting the appendicular skeleton [5], neurological disorders [6]. In some dogs with chronic *B. canis* infection, the onset of recurrent anterior uveitis with corneal edema has also been described [7,8]. Other manifestations of *B. canis* infection may be represented by polyarthritis [6].

*B. canis* is considered endemic in several regions, primarily due to limited control measures, underreporting, and the absence of mandatory surveillance programs [1]. Brucellosis in dogs occurs worldwide and is endemic to the Americas, Asia, Africa [9], and Europe [1]. However, the global distribution is likely underestimated, as many countries lack systematic testing or reporting requirements, particularly for companion animals and imported dogs. This patchy surveillance contributes to uncertainty in defining true endemic areas and underscores the need for coordinated international monitoring and standardized diagnostic protocols [10].

Humans in close contact with infected dogs, particularly immunocompromised individuals, face a potential risk of infection [1].

Despite its importance, *B. canis* remains underdiagnosed due to the lack of standardized testing and control measures. The disease’s insidious nature, often presenting with nonspecific or asymptomatic infections, complicates diagnosis and containment efforts. In Europe, sporadic outbreaks have been reported, but widespread surveillance programs are still absent 13]. This lack of regulatory oversight allows for continued silent transmission, particularly through unregulated international dog trade and breeding practices [11].

Currently, no globally standardized protocol exists for *B. canis* diagnosis, control, and treatment [12]. Antibiotic therapy has a high relapse rate, making complete eradication challenging, and castration alone may not fully eliminate bacterial reservoirs [13]. Given the increasing international movement of dogs, particularly from endemic regions, the lack of systematic screening presents a significant risk for disease transmission [14].

In Italy, several surveys reported the presence of seropositivity at various levels and in different geographical areas [15]. Corrente *et al*., [16] detected *B. canis* by PCR in a half-breed dog suffering from chronic prostatitis and discospondylitis. In 2020, *B. canis* has been detected and isolated for the first time in a commercial breeding kennel [15]. This study details the protocol developed in response to Italy’s first *B. canis* outbreak, focusing on non-infected animal identification and rehoming as a strategy for outbreak containment. By analyzing the outcomes of this approach, we aim to highlight best practices in disease management while advocating for more comprehensive national and international regulatory measures.

## 2 Materials and methods

### 2.1 Outbreak identification and initial response

In April 2020, Italy recorded its first *B. canis* outbreak. Prior to this event, serological cases had been sporadically reported, but the bacterium had never been isolated. The outbreak occurred in a large breeding kennel in central Italy that housed mainly Chihuahuas along with other toy breeds such as Pomeranian Spitz, Maltese, and Toy Poodle [15].

Serological analyses were performed using a microplate serum agglutination test (mSAT), adapted from the tube agglutination method by Alton et al. [17] for use in 96-well U-shaped plates. *B. canis* strain RM66 was used to prepare the antigen. Serum samples were diluted 1:10 in Tris-maleate buffer (pH 9.0 ± 0.5), then serially diluted two-fold in the microplate with equal volumes of antigen and serum (50 μl each), resulting in final dilutions from 1:20–1:640. Plates were incubated at 37 ± 2 °C for 48 hours. Samples showing 100% agglutination at dilutions ≥1:20 were considered positive, with titers reported as the highest dilution showing complete agglutination [15].

Blood and urine samples were streaked onto selective Farrell’s *Brucella* Agar and inoculated into enrichment broth with equine serum. All cultures were incubated at 37 ± 1 °C. Broth cultures were subcultured weekly onto Farrell’s *Brucella* Agar for one month. Plates were checked daily for growth and considered negative if no colonies appeared in direct or subcultures. Suspected *Brucella* colonies were confirmed by species-specific PCR [15,18].

### 2.2 Implementation of the No-Kill strategy

Given the widespread public concern in Italy regarding pet welfare, euthanasia was not considered an option. In fact, it is prohibited by national law unless legally justified such as in cases of terminally illness or dangerous animals. Instead, a no-kill strategy was adopted, despite the significant logistical and financial challenges it posed, particularly due to the large number of animals involved and the substantial resources required for their care.

Following the confirmation of *B. canis* in the kennel, strict movement restrictions and a ban on sales were implemented to prevent further disease spread. These measures applied to both external transactions and internal transfers within the kennel. Additionally, male and female dogs were separated to prevent further breeding, reduce the risk of abortions, and minimize pathogen release.

### 2.3 Dog population control and testing protocol

To control the outbreak while managing the kennel population, a structured protocol combining neutering and castration and systematic testing was implemented. However, due to the frequent colonization of *B. canis* in the prostate of male dogs, which leads to bacterial shedding through urine, efforts to select negative animals eligible for rehoming were primarily focused on female dogs. Subsequently, the same protocol was applied to a restricted number of male castrated dogs that remained constantly negative since the beginning of the outbreak.

Female dogs underwent neutering, together with primary serological and bacteriological testing. Laboratory analyses were carried as previously described [15,19]. Concerning serological tests, seronegative status was determined using mSAT, with a cut-off titre of 1:20; dogs with titres below this threshold were classified as seronegative. The diagnostic performance of the mSAT, as validated by Perletta *et al.* [19], indicates an estimated sensitivity of 96.7% (88.8–98.7) and a specificity of 92.3% (86.7–95.1).

Simultaneously, all male dogs underwent castration and animals testing negative to serological and bacteriological screening carried out during outbreak investigations were considered for rehoming and followed the same protocol of female dogs with an additional bacteriological control on urine samples. Animals testing negative to serological and bacteriological investigations were classified as negative and eligible for adoption.

### 2.4 Seronegative animals selected for potential rehoming

Firstly, seronegative dogs were transferred to a separate facility under quarantine conditions. Then, sequential testing in quarantine has been done, as follows: both serological and bacteriological tests were performed at day 10, in a first round of quarantine; at day 28 only serological testing was conducted and an additional bacteriological testing was carried out on urine samples only for male dogs (second round); in the final evaluation round (week 8) dogs that remained negative throughout all testing phases were cleared for adoption. Furthermore, dogs testing positive at any stage were returned to the original outbreak kennel for further management.

The testing intervals (days 10, 28, and 8 weeks) were selected based on (i) the known kinetics of *B. canis* infection, including the typical window for seroconversion; (ii) the intermittent nature of bacteraemia and urinary shedding; and (iii) the need to balance diagnostic accuracy with feasible management of a large canine population. The day 10 assessment allowed early detection of dogs that might have been incubating the infection earlier; the day 28 testing corresponded to the expected timeframe for seroconversion in animals exposed shortly before the start of quarantine; and the final evaluation at 8 weeks provided a sufficient temporal buffer to identify delayed serological or bacteriological positives before rehoming for infections acquired around the 28^th^ day of testing (but still silent at the 28^th^ day’s sampling). These intervals were therefore chosen to minimize the risk of releasing dogs with early, subclinical, or intermittently detectable infections.

A small proportion of dogs tested positive during the first and second testing rounds and were consequently removed from the rehoming pathway and returned to the outbreak facility for continued management. No dogs that remained negative through all testing rounds converted at later timepoints.

The outbreak kennel remained under official movement restrictions. These dogs continued to be housed at the facility under veterinary supervision, following national guidelines for infectious disease containment. Due to the absence of a standardized treatment protocol and the high relapse rate associated with antibiotic therapy, no further rehoming of positive animals was permitted. Their long-term management remained under the control of the local veterinary authority.

Dogs were kept in a dedicated, physically separated facility under strict biosecurity measures. Housing adhered to national animal-welfare standards, ensuring adequate space, environmental enrichment, climate control, and daily monitoring by veterinary staff. Additionally, caretakers used individualized equipment and PPE to reduce cross-contamination risks. These details are now included to provide assurance that the quarantine procedures safeguarded both animal well-being and biosafety.

**Fig 1:**
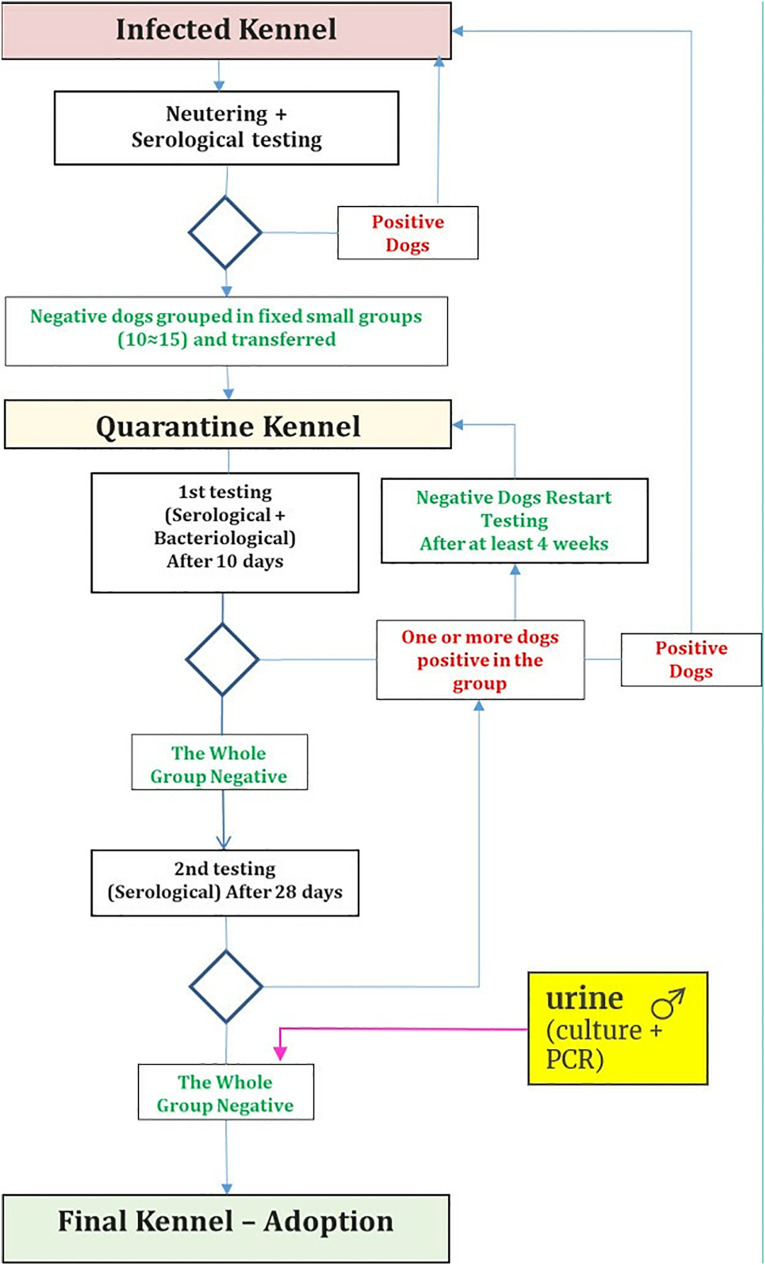
Rehoming testing strategy flow chart.

Fig 1 summarizes the flow of our rehoming testing strategy.

### 2.5 Post-adoption monitoring and owner responsibilities

To ensure long-term health monitoring, all adopted dogs were subjected to post-adoption testing at defined intervals:

- Three months post-adoption
- Six months post-adoption
- Twelve months post-adoption
- Annually thereafter (recommended for life)

Adoptive owners were required to sign a consent and awareness form outlining the risks associated with adopting a dog from an outbreak setting. This document provided a comprehensive guidance on preventive measures, hygiene protocols, and importance of continued testing. Owners were also responsible for covering the costs of post-adoption testing, reinforcing their critical role in maintaining effective disease control.

### 2.6 Ethical, legal, and animal welfare considerations

All procedures involving animals were conducted in accordance with national and institutional guidelines for the care and use of laboratory animals. This study was carried out under the supervision of the official veterinary services and adhered to Italian legislation prohibiting euthanasia unless medically or legally justified. Informed consent was obtained from all adoptive owners prior to inclusion in the post-adoption monitoring program.

## 3 Results

Of the 683 dogs tested (475 females, 69.5%; 208 males, 30.5%), 241 were positive by mSAT, and 68 were positive by bacterial culture. Among these, 64 dogs tested positive by both mSAT and bacterial isolation, 177 only in mSAT, and 4 dogs only by culture. This results in a total of 245 dogs (35.9%) testing positive by at least one method. The remaining 438 dogs (64.1%) tested negative in both tests, as described by De Massis *et al*. [15] (Table 1).

**Table 1:** Comparison of positive and negative results obtained by *B. canis* microplate serum agglutination test (mSAT) and *Brucella* spp. isolation method.

		Serology (mSAT)				
		Positive	Negative	Total		
**Isolation**	Positive	64	4	68	94,1%	PPV
	Negative	177	438	615	71,2%	NPV
	Total	241	442	683		

As of October, 25th, 2023, a total of 409 dogs had been successfully rehomed (56.4% females and 43.6% males), and 315 dogs (77.0%) had undergone follow-up testing. Importantly, all tested dogs remained negative for *B. canis* in both serological and bacteriological assessments.

**Fig 2:**
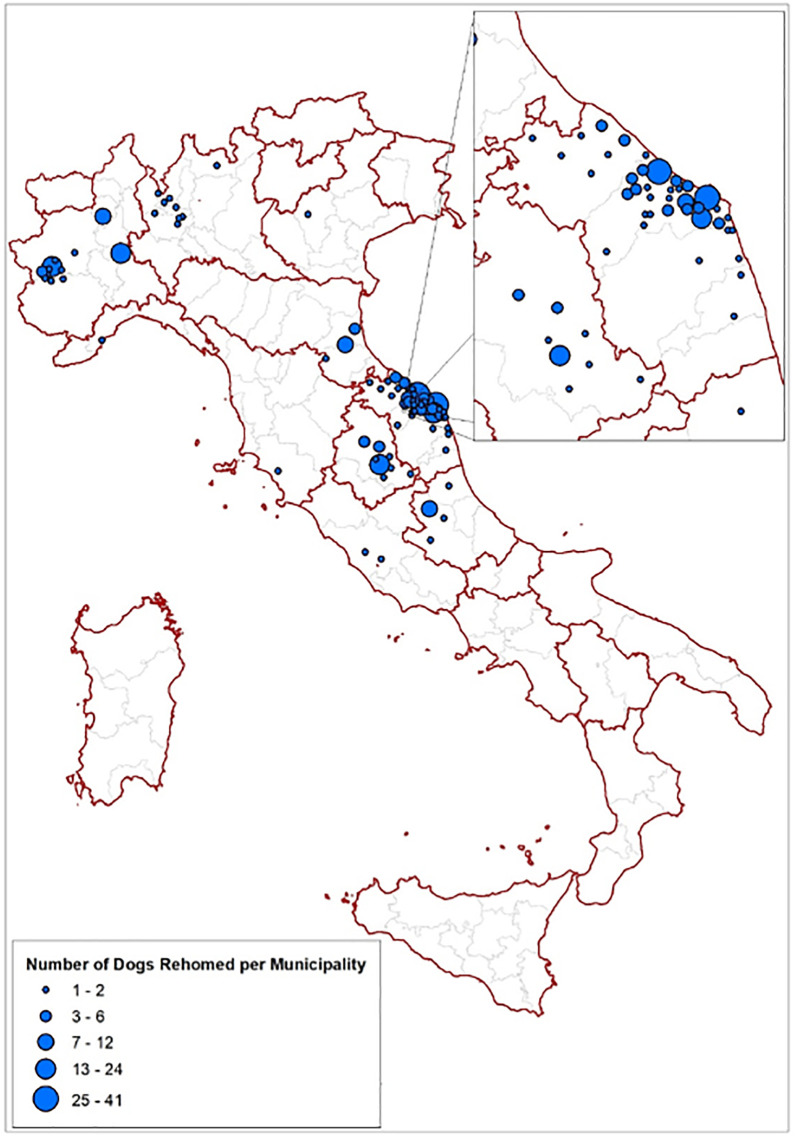
Number of dogs rehomed per municipality.

**Fig 3:**
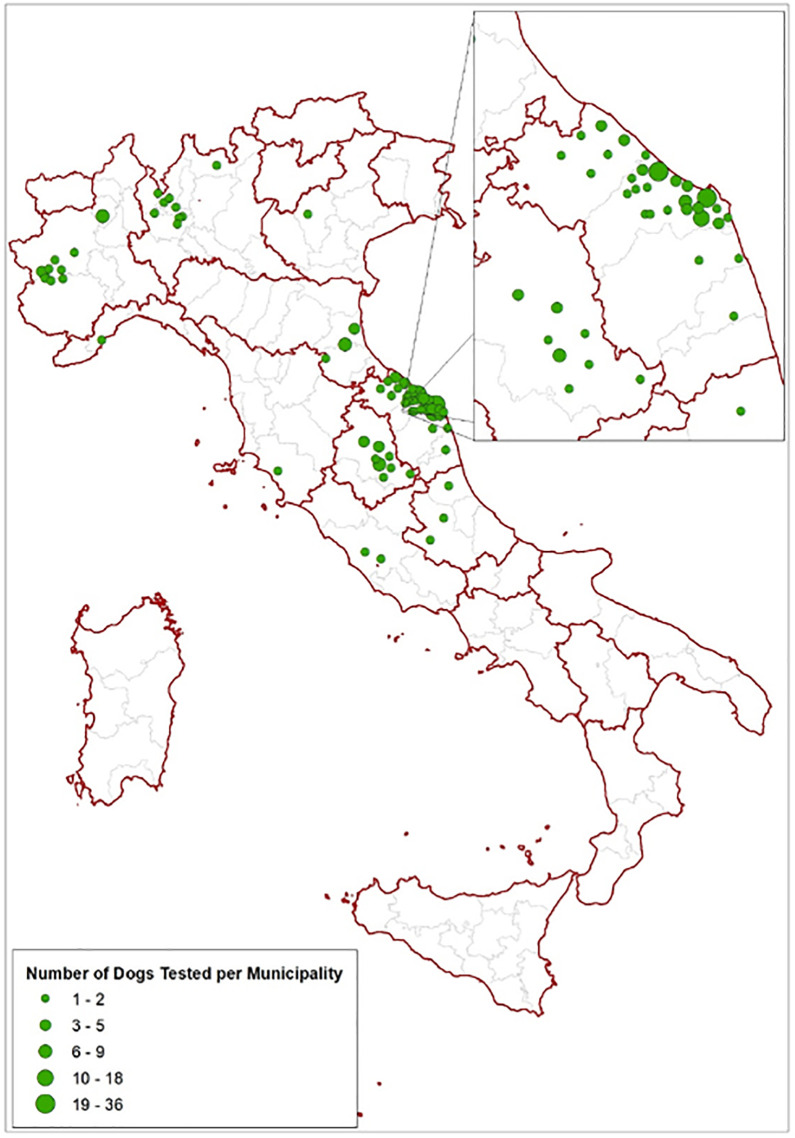
Number of dogs tested per municipality.

Figs 2 and 3 illustrate the distribution of rehomed dogs and tested dogs per municipality, respectively. The data indicate that the rehoming strategy was successfully implemented across multiple regions, with consistently negative test results validating the reliability of the protocol.

In addition to test outcomes, basic demographic characteristics of the population were examined to provide a clearer epidemiological profile. Among the 683 dogs tested, the population consisted predominantly of toy breeds, reflecting the composition of the affected kennel. Of the 208 male (30.5%) and 475 (69.5%) female dogs, the distribution of seropositivity to the mSAT test was different between males and females. Overall, 48 males were positive out of 208 (23.1%), while 193 females were positive out of 475 (40.6%; Table 2). This difference was found as significant (χ2 = 19.52; p < 0.01) (De Massis *et al.*, 2021).

**Table 2:** Comparison of positive and negative results obtained by *B. canis* microplate serum agglutination test method (mSAT) in males and females.

Serology (mSAT)	Negative	Positive	Grand Total	
Females	282	193	475	40,6%
Males	160	48	208	23,1%
Grand Total	442	241	683	35,3%
	χ^2^=	19,52194		
	P=	0,000010	Significant (P < 0.01)	

Of the 208 male (30.5%) and 420 (69.5%) female dogs, the distribution of positivity to bacteriology was again different between males and females. Overall, 13 males were positive out of 195 (6.3%), while 55 females were positive out of 420 (11.6%; Table 3). This difference was found as significant (χ2 = 4.58; p < 0.05) (De Massis *et al.*, 2021).

**Table 3:** Comparison of positive and negative results obtained by *B. canis* isolation method in males and females.

Bacteriology	Negative	Positive	Grand Total		
Females	420	55	475	11,6%	
Males	195	13	208	6,3%	
Grand Total	615	68	683	10,0%	
	χ^2^=	4,582226			
	P=	0,032305	Significant (P < 0.05)		

Age distribution ranged from days to 8.5 years of age for the oldest dog. The dogs in the kennel were 2.8 years old in average, and both positive and negative dogs showed similar age profiles, indicating that infection was not concentrated in a specific age class (De Massis *et al.*, 2021).

## 4 Discussion

### 4.1 Validation of the No-Kill strategy

To the best of our knowledge, this is the first large-scale attempt at managing a *B. canis* outbreak in a breeding kennel through a no-kill strategy. The absence of infection in rehomed dogs supports the effectiveness of this approach. This aligns with findings from smaller-scale studies, which suggest that a combination of castration and neutering and rigorous screening can mitigate transmission risks [20]. In some cases, low seropositivity titers, close to test cut-off values (1:20 dilution for mSAT) were detected during initial post-adoption screenings. However, these cases were not confirmed in following testing, and all affected dogs consistently tested negative in bacterial isolation, suggesting that the observed low titers were likely nonspecific or transient responses rather than active infections.

### 4.2 Comparison with international outbreak responses

Unlike outbreaks in other countries where euthanasia has been a primary response, our strategy demonstrates that rehoming non-infected animals can be a viable alternative. [21]. The recommended UK approach for confirmed infected animals is euthanasia, however for test-positive clinically normal dogs antimicrobial therapy and neutering could be a solution, combined with a limitation in contact with other dogs and further hygiene measures for the rest of the dog’s life. [22]. In Netherlands euthanasia is the only approach for seropositive dogs and dogs they were in close contact with, while a periodically testing (6 months) is chosen for the seronegative remaining dogs [23]. The Canadian generally accepted recommendation is that truly infected dogs should be euthanized due to the risk to canine and human populations. However, when euthanasia is not possible, due to client opinion, then isolation can be considered after appropriate client education and medical record documentation [24]. In the U.S., delays in veterinary responses to multi-state outbreaks highlighted the need for standardized control measures [21]. European outbreaks similarly lacked coordinated response protocols, often relying on ad hoc interventions [25].

Our findings suggest that structured rehoming programs can serve as a humane and effective disease management tool, and demonstrate the effectiveness of the structured rehoming and monitoring protocol, ensuring that no cases of late-onset *B. canis* infection have emerged to date. The results support the viability of controlled adoption as a strategy for managing *B. canis* outbreaks while adhering to ethical and legal constraints.

While our approach demonstrated success, it remains contingent on rigorous follow-up testing and owner compliance. In scenarios where resources or owner adherence are insufficient, alternative containment strategies may need to be explored. For example, future studies should investigate the role of long-term antibody monitoring and the feasibility of a vaccination program. Additionally, genomic sequencing of *B. canis* strains involved in outbreaks could provide critical epidemiological insights and improve tracking of disease spread across borders [26].

### 4.3 Challenges in surveillance and control

The lack of a globally standardized protocol for *B. canis* control creates persistent challenges in disease management and prevention. In Europe, dogs are frequently imported without mandatory pre- or post-arrival testing, making surveillance difficult [10]. Even within Italy, fragmented canine registry system further complicates trace-forward investigations during outbreaks. Establishing mandatory identification and health certification protocols for imported dogs could significantly enhance disease tracking and prevention.

The reluctance of some pet owners to comply with follow-up testing requirements further complicates monitoring efforts. In some cases, the absence of legal enforcement mechanisms prevents veterinary authorities from compelling compliance. Future policies should address these gaps by integrating owner responsibility clauses and government-funded testing incentives for high-risk animals [25].

### 4.4 Policy and one health implications

Given the zoonotic potential of *B. canis*, a One Health approach is crucial for controlling its spread. Recommendations include:

- Mandatory pre-import screening for dogs entering non-endemic regions.
- Standardized testing protocols for breeders and veterinary clinics.
- Owner education initiatives to ensure proper monitoring and reporting.
- Further research into antimicrobial treatments and vaccine development [27].

The ethical considerations surrounding euthanasia versus treatment should also be addressed. While antimicrobial therapy remains inconsistent, euthanasia faces resistance from both the public and animal welfare organizations. Future guidelines must balance disease control with ethical concerns [26].

### 4.5 Study limitations

Despite the structured nature of the rehoming and monitoring protocol, the approach is not without limitations. *Brucella canis*, often referred to as “the great imposter,” poses a significant diagnostic challenge due to its potential for asymptomatic carriage and intermittent bacterial shedding. This, combined with imperfect test sensitivity and specificity, necessitates cautious interpretation of negative results.

A critical limitation encountered in our study was the approximately 20% loss to follow-up among rehomed dogs. Although adopters were informed of the need for continued testing and signed consent forms acknowledging their responsibilities, enforcement of long-term compliance remains a challenge in the absence of binding legal mechanisms. This gap introduces a residual risk of undetected transmission in the community.

Dogs that lost to follow-up may not be representative of those who completed all scheduled post-adoption tests. These animals could differ in owner compliance, access to veterinary care, or underlying health conditions, introducing the possibility that undetected late infections occurred outside the monitoring framework. Although all rehomed dogs underwent a rigorous multi-step testing protocol before adoption, this attrition still represents a source of uncertainty that may modestly affect the estimated reliability of the rehoming strategy.

Because *B. canis* is characterized by intermittent shedding and variable antibody responses, negative results, even when repeated, cannot absolutely exclude infection. We highlight that this limitation necessitates cautious interpretation of our findings and underscores the importance of continued long-term monitoring and the development of more sensitive diagnostic methods.

Et al.To mitigate such risks, only dogs that remained seronegative throughout multiple testing phases were deemed eligible for adoption. Furthermore, we recommend that future implementations of this strategy be accompanied by policy enhancements such as mandatory post-adoption testing requirements, government-funded diagnostic programs, and integration of health status monitoring via national microchip registries.

## 5 Conclusion

Considering the increasing movement of companion animals, implementing robust identification and registration systems is essential for effective epidemiological surveillance. Our findings indicate that rehoming non-infected dogs, when combined with a structured screening protocol, is a viable alternative to mass euthanasia. Moreover, a standardized international approach to *B. canis* screening and outbreak response is urgently needed, while stronger regulations on dog importation and movement could reduce transmission risks.

Further research is needed to develop effective vaccination strategies, improve diagnostic test sensitivity, and establish international policy frameworks to regulate the movement of dogs from endemic regions. Surveillance systems should be expanded, and veterinary authorities must collaborate globally to prevent future outbreaks.

Ultimately, a One Health strategy—integrating veterinary, public health, and policy measures—is necessary to ensure effective control and long-term management of *B. canis* outbreaks. The success of this approach in Italy provides a model that can be adapted for use in other regions facing similar epidemiological threats.

## References

[1] Djokic V, Freddi L, de Massis F, Lahti E, van den Esker MH, Whatmore A, et al. The emergence of Brucella canis as a public health threat in Europe: what we know and what we need to learn. Emerg Microbes Infect. 2023;12(2):2249126. doi: 10.1080/22221751.2023.2249126 37649455 PMC10540651

[2] Tymczak M, Favi B, Beccaglia M, Pisu MC, Tarducci V, Franciosini MP, et al. Are Italian-Polish veterinarians and breeders prepared to control an outbreak of Brucella canis infection in dogs? Pol J Vet Sci. 2022;25(3):411–8. doi: 10.24425/pjvs.2022.142025 36155554

[3] Santos RL, Souza TD, Mol JPS, Eckstein C, Paíxão TA. Canine brucellosis: an update. Front Vet Sci. 2021;8:594291. doi: 10.3389/fvets.2021.594291 33738302 PMC7962550

[4] Henderson RA, Hoerlein BF, Kramer TT, Meyer ME. Discospondylitis in three dogs infected with Brucella canis. J Am Vet Med Assoc. 1974;165(5):451–5. doi: 10.2460/javma.1974.165.05.451 4423220

[5] Smeak DD, Olmstead ML, Hohn RB. Brucella canis osteomyelitis in two dogs with total hip replacements. J Am Vet Med Assoc. 1987;191(8):986–90. doi: 10.2460/javma.1987.191.08.986 3679997

[6] Carmichael LE. Canine brucellosis. In: Infectious diseases of the dog and cat (Greene C.E., ed) 4th Ed. London: Elsevier Health Sciences; 2012. p. 398–411.

[7] Saegusa J, Ueda K, Goto Y, Fujiwara K. Ocular lesions in experimental canine brucellosis. Nihon Juigaku Zasshi. 1977;39(2):181–5. doi: 10.1292/jvms1939.39.181 559214

[8] Riecke JA, Rhoades HE. Brucella canis isolated from the eye of a dog. J Am Vet Med Assoc. 1975;166(6):583–4. doi: 10.2460/javma.1975.166.06.583 1120729

[9] Sebzda MK, Kauffman LK. Update on Brucella canis: Understanding the Past and Preparing for the Future. Vet Clin North Am Small Anim Pract. 2023;53(5):1047–62. doi: 10.1016/j.cvsm.2023.05.002 37385876

[10] Keid LB, Soares RM, Vasconcellos SA, Chiebao DP, Megid J, Salgado VR, et al. A polymerase chain reaction for the detection of Brucella canis in semen of naturally infected dogs. Theriogenology. 2007;67(7):1203–10. doi: 10.1016/j.theriogenology.2007.01.003 17343907

[11] Johnson CA, Carter TD, Dunn JR, Baer SR, Schalow MM, Bellay YM, et al. Investigation and characterization of Brucella canis infections in pet-quality dogs and associated human exposures during a 2007-2016 outbreak in Michigan. J Am Vet Med Assoc. 2018;253(3):322–36. doi: 10.2460/javma.253.3.322 30020006 PMC6642745

[12] Wanke MM. Canine brucellosis. Anim Reprod Sci. 2004;82–83:195–207. doi: 10.1016/j.anireprosci.2004.05.005 15271453

[13] De Massis F, Sacchini F, Averaimo D, Garofolo G, Lecchini P, Ruocco L, et al. First Isolation of Brucella canis from a breeding kennel in Italy. Vet Ital. 2021;57:3. doi: 10.12834/VetIt.2497.15848.1 34641664

[14] Corrente M, Franchini D, Decaro N, Greco G, D’Abramo M, Greco MF, et al. Detection of Brucella canis in a dog in Italy. New Microbiol. 2010;33(4):337–41. 21213592

[15] Alton GG, Jones LM, Angus RD, Verger JM. Techniques for the brucellosis laboratory. Paris: I.N.R.A.; 1988. p. 169–74.

[16] World Organization for Animal Health (OIE). Brucellosis (*Brucella abortus*, *B. melitensis* and *B. suis*) (infection with *B. abortus*, *B. melitensis* and *B. suis*) In Manual of Diagnostic Tests and Vaccines far Terrestrial Animals. Paris: Office International des Epizooties; 2019. p. 1–44.

[17] Perletta F, Di Pancrazio C, Rodomonti D, Di Febo T, Luciani M, Krasteva IM, et al. Evaluation of three serological tests for diagnosis of canine brucellosis. Microorganisms. 2023;11(9):2162. doi: 10.3390/microorganisms11092162 37764006 PMC10536495

[18] Ledbetter EC, Landry MP, Nowowiejski S, Kern TJ. Ocular manifestations of *Brucella canis* infection in dogs: 11 cases. J Am Vet Med Assoc. 2009;234(9):1165–70. doi: 10.2460/javma.234.9.1165 19405987

[19] Middlemiss C. Brucella canis in dogs in the UK. Vet Rec. 2021;188(4):155. doi: 10.1002/vetr.227 34651711

[20] Kolwijck E, Lutgens SPM, Visser VXN, van Apeldoorn MJ, Graham H, Koets AP, et al. First Case of Human *Brucella canis* Infection in the Netherlands. Clin Infect Dis. 2022;75(12):2250–2. doi: 10.1093/cid/ciac425 35653425

[21] Hensel ME, Negron M, Arenas-Gamboa AM. Brucellosis in Dogs and Public Health Risk. Emerg Infect Dis. 2018;24(8):1401–6. doi: 10.3201/eid2408.171171 30014831 PMC6056133

[22] Baek BK, Lim CW, Rahman MS, Kim C-H, Oluoch A, Kakoma I. *Brucella abortus* infection in indigenous Korean dogs. Can J Vet Res. 2003;67(4):312–4. 14620870 PMC280718

[23] Carmichael LE, Joubert JC. Transmission of Brucella canis by contact exposure. Cornell Vet. 1988;78(1):63–73. 3335131

[24] De Massis F, Sacchini F, Petrini A, Bellucci F, Perilli M, Garofolo G, et al. Canine brucellosis due to Brucella canis: description of the disease and control measures. Vet Ital. 2022;58(1):5–23. doi: 10.12834/VetIt.2561.16874.1 35766163

[25] Marzetti S, Carranza C, Roncallo M, Escobar GI, Lucero NE. Recent trends in human *Brucella canis* infection. Comp Immunol Microbiol Infect Dis. 2013;36(1):55–61. doi: 10.1016/j.cimid.2012.09.002 23040615

[26] Kauffman LK, Petersen CA. Canine brucellosis: New insights into diagnosis, treatment, and prevention. J Vet Intern Med. 2019;33(2):1965–75. doi: 10.1111/jvim.15621 31692600

[27] Cosford KL. Brucella canis: An update on research and clinical management. Can Vet J. 2018;59(1):74–81. 29302106 PMC5731389

