# Supplementary material for: Brucella canis outbreak management towards rehoming strategies: The Italian experience
Source: PLoS One. 2026 Jun 30;21(6):e0351742. doi: 10.1371/journal.pone.0351742 (PMC13318041; doi:10.1371/journal.pone.0351742)
Supplement: Attachment — Submitted filename: Reply to the Editor on Data disclosing.docx [file pone.0351742.s002.docx]

**Subject: Further clarification of legal restrictions on data sharing**

Dear Editor,

Thank you for the opportunity to further clarify the legal restrictions preventing public sharing of the data used in our study.

The dataset analyzed in this manuscript derives from administrative health records held by a Local Health Unit (Azienda Sanitaria Territoriale, AST) in Italy. These data are owned by the AST and are classified as personal health data under Regulation (EU) 2016/679 (General Data Protection Regulation, GDPR) and Italian national legislation (Legislative Decree No. 196/2003, as amended by Legislative Decree No. 101/2018).

Under the data use agreement and authorization granted to the authors, access to the individual-level data is strictly limited to the approved research project. The AST explicitly prohibits public dissemination, redistribution, or deposition of the raw data in external repositories, even in de-identified form. This restriction is due to:

- the sensitive nature of health-related information (*e.g.* the points that could be extracted from images, *i.e.* the geographical coordinates, could explicitly make it possible to identify private houses positions);
- the potential risk of re-identification when combining variables at the individual level (i.e. owner sensitive information associated to dog microchips could be retrieved in the National I&R database for pets
- the legal obligation of the data controller (the ASL) to prevent secondary use or disclosure beyond the authorized purposes.

The legal basis for data processing allows analysis and publication of results only in aggregated and anonymized form. Accordingly, all results in the manuscript are reported at an aggregate level, and no individual-level data are disclosed.

Researchers who meet the legal and ethical requirements for access to confidential health data may submit a formal data access request to the data-owning authority:

Dipartimento di Prevenzione AST Ancona, Servizio Sanità Animale, Via C. Colombo, 106 (Ancona), 61100 Ancona (AN) - Italy.

Office or Department responsible for data governance / privacy: urp.ast.an@sanita.marche.it.

Institutional contact email or official website: direzionegenerale.ast.an@sanita.marche.it

We believe these constraints fall under the ethical and legal exceptions described in the PLOS Data Availability Policy. Within these limits, we have ensured full transparency of methods, analyses, and aggregated results to allow reproducibility to the maximum extent permitted by law.

Please let us know if further clarification or documentation is required.

Sincerely,

Nausica D’Aurelio

on behalf of all authors

Istituto Zooprofilattico Sperimentale del’Abruzzo e del Molise “G. Caporale”

Branch Veterinary Laboratory of Pescara.

Via Raiale 118/2

65124 Pescara - Italy
