# Supplementary material for: Brucella canis outbreak management towards rehoming strategies: The Italian experience
Source: PLoS One. 2026 Jun 30;21(6):e0351742. doi: 10.1371/journal.pone.0351742 (PMC13318041; doi:10.1371/journal.pone.0351742)
Supplement: Attachment — Submitted filename: Rebuttal letter_05.docx [file pone.0351742.s003.docx]

Dear Reviewers,

We would like to sincerely thank you for your careful review and constructive feedback on our manuscript. Your comments have helped us improve the clarity, accuracy, and overall quality of our work. Please find our detailed point-by-point responses below.

**Reviewer #1**

The authors have addressed the reviewer comments thoroughly and substantially improved the manuscript, particularly in the Methods, Results, and Limitations sections. The study is well-structured and provides valuable insight into B. canis outbreak management. A few minor grammatical issues still remain. Pending these minor revisions, I recommend acceptance.

***Comment:*** *1)* "discospondlytis”: should be “discospondylitis

***Response***: Thank you for this comment, we corrected the term.

**Comment**: 2) Bracket error: “Africa 12], and Europe [1]”

***Response****: Thank you, we added the bracket.*

**Comment**: 3) Miss space after "programs: "absence of mandatory surveillance programs[1]"

***Response****: Thank you for this observation, we added the space after “programs”.*

**Comment**: 4) This sentence appears twice so need to delete one instance: “Because B. canis is characterized by intermittent shedding…”

**Response**: Thank you for the comment, we deleted the second one.

**Comment**: 5) Broken/fragmented sentence and not sure where the et al was suppose to go to: "In 2020, B. canis has been detected and isolated for the first time in a commercial breeding kennel [18]. et al."

**Response**: Thank you for the observation, that “et al.” was a typo, we deleted it.

**Reviewer #2**

Thank you for your careful revision and detailed response. The manuscript is substantially improved in clarity and methodological transparency. Most of my prior concerns have been addressed; just a few edits are required.

Point-by-point assessment relative to my previous review:

Major comments

Lines 40 to 51: Insert the references where appropriate for easier identification of which reference supports which statement.

- Assessment: The authors included references for each statement.

Table 1: As there is a clear difference in management between males and female dogs having the test results by sex would provide more information to the reader.

- Assessment: The authors have included additional tables which provide a breakdown of the serological and culture results per sex for better understanding.

Minor comments

***Comment:*** Line 59: It seems that the sentence starts with a “et al.” which does not seem connected to any sentence. It needs to be removed.

***Response:*** Thank you for this observation. As written previously, that “et al.” was a typo, we deleted it.

***Comment:*** Table 1: The first line of the table reads “Serology (Msat)” where it should read “Serology (mSAT)”. Please make the appropriate change. Review Comments to the Author

***Response:*** Thank you for the comment, we changed it.
