# Supplementary material for: Brucella canis outbreak management towards rehoming strategies: The Italian experience
Source: PLoS One. 2026 Jun 30;21(6):e0351742. doi: 10.1371/journal.pone.0351742 (PMC13318041; doi:10.1371/journal.pone.0351742)
Supplement: Peer review history

# Peer review: Decision Letter 0

**DOI:** 10.1371/journal.pone.0351742.r001

**Authors:** Roy Roop

14 Sep 2025

PONE-D-25-43850

*Brucella canis*  Outbreak Managment Towards Rehoming Strategies: The Italian Experience

PLOS ONE

Dear Dr. D'Aurelio,

Thank you for submitting your manuscript to *PLOS ONE* . Two expert reviewers evaluated your paper and their comments are provided below. Both expressed the opinion that the information provided represents an important contribution to the field. But both also pointed out some issues that need to be addressed before the paper will be considered acceptable for publication in the journal. Consequently, I am going to ask that you submit a revised version and adequately and appropriately addresses all of  the comments raised by both reviewers.

Please submit your revised manuscript by Oct 29 2025 11:59PM. If you will need more time than this to complete your revisions, please reply to this message or contact the journal office at plosone@plos.org. When you're ready to submit your revision, log on to https://www.editorialmanager.com/pone/ and select the 'Submissions Needing Revision' folder to locate your manuscript file.

Please include the following items when submitting your revised manuscript:

- A rebuttal letter that responds to each point raised by the academic editor and reviewer(s). You should upload this letter as a separate file labeled 'Response to Reviewers'.
- A marked-up copy of your manuscript that highlights changes made to the original version. You should upload this as a separate file labeled 'Revised Manuscript with Track Changes'.
- An unmarked version of your revised paper without tracked changes. You should upload this as a separate file labeled 'Manuscript'.

-->If you would like to make changes to your financial disclosure, please include your updated statement in your cover letter. Guidelines for resubmitting your figure files are available below the reviewer comments at the end of this letter.

If applicable, we recommend that you deposit your laboratory protocols in protocols.io to enhance the reproducibility of your results. Protocols.io assigns your protocol its own identifier (DOI) so that it can be cited independently in the future. For instructions see: https://journals.plos.org/plosone/s/submission-guidelines#loc-laboratory-protocols. Additionally, PLOS ONE offers an option for publishing peer-reviewed Lab Protocol articles, which describe protocols hosted on protocols.io. Read more information on sharing protocols at https://plos.org/protocols?utm_medium=editorial-email&utm_source=authorletters&utm_campaign=protocols.

We look forward to receiving your revised manuscript!

Sincerely,

Marty Roop

Academic Editor

*PLOS ONE*

Journal Requirements:

When submitting your revision, we need you to address these additional requirements.

1. Please ensure that your manuscript meets PLOS ONE's style requirements, including those for file naming. The PLOS ONE style templates can be found at https://journals.plos.org/plosone/s/file?id=wjVg/PLOSOne_formatting_sample_main_body.pdf and https://journals.plos.org/plosone/s/file?id=ba62/PLOSOne_formatting_sample_title_authors_affiliations.pdf

2. We noticed you have some minor occurrence of overlapping text with the following previous publication(s), which needs to be addressed:

- doi: 10.12834/VetIt.2561.16874.1

In your revision ensure you cite all your sources (including your own works), and quote or rephrase any duplicated text outside the methods section. Further consideration is dependent on these concerns being addressed.

3. Your ethics statement should only appear in the Methods section of your manuscript. If your ethics statement is written in any section besides the Methods, please move it to the Methods section and delete it from any other section. Please ensure that your ethics statement is included in your manuscript, as the ethics statement entered into the online submission form will not be published alongside your manuscript.

4. We note that your Data Availability Statement is currently as follows: All relevant data are within the manuscript and its Supporting Information files.

Please confirm at this time whether or not your submission contains all raw data required to replicate the results of your study. Authors must share the “minimal data set” for their submission. PLOS defines the minimal data set to consist of the data required to replicate all study findings reported in the article, as well as related metadata and methods (https://journals.plos.org/plosone/s/data-availability#loc-minimal-data-set-definition).

For example, authors should submit the following data:

- The values behind the means, standard deviations and other measures reported;

- The values used to build graphs;

- The points extracted from images for analysis.

Authors do not need to submit their entire data set if only a portion of the data was used in the reported study.

If your submission does not contain these data, please either upload them as Supporting Information files or deposit them to a stable, public repository and provide us with the relevant URLs, DOIs, or accession numbers. For a list of recommended repositories, please see https://journals.plos.org/plosone/s/recommended-repositories.

If there are ethical or legal restrictions on sharing a de-identified data set, please explain them in detail (e.g., data contain potentially sensitive information, data are owned by a third-party organization, etc.) and who has imposed them (e.g., an ethics committee). Please also provide contact information for a data access committee, ethics committee, or other institutional body to which data requests may be sent. If data are owned by a third party, please indicate how others may request data access.

5. Please note that funding information should not appear in any section or other areas of your manuscript. We will only publish funding information present in the Funding Statement section of the online submission form. Please remove any funding-related text from the manuscript.

6. Please amend either the title on the online submission form (via Edit Submission) or the title in the manuscript so that they are identical.

7. Please ensure that you refer to Figure 1, 2, and 3 in your text as, if accepted, production will need this reference to link the reader to the figure.

8. We note that Figure(s) 2 and 3 in your submission contain [map/satellite] images which may be copyrighted. All PLOS content is published under the Creative Commons Attribution License (CC BY 4.0), which means that the manuscript, images, and Supporting Information files will be freely available online, and any third party is permitted to access, download, copy, distribute, and use these materials in any way, even commercially, with proper attribution. For these reasons, we cannot publish previously copyrighted maps or satellite images created using proprietary data, such as Google software (Google Maps, Street View, and Earth). For more information, see our copyright guidelines: http://journals.plos.org/plosone/s/licenses-and-copyright.

We require you to either (1) present written permission from the copyright holder to publish these figures specifically under the CC BY 4.0 license, or (2) remove the figures from your submission:

a. You may seek permission from the original copyright holder of Figure(s) 2 and 3 to publish the content specifically under the CC BY 4.0 license.

We recommend that you contact the original copyright holder with the Content Permission Form (http://journals.plos.org/plosone/s/file?id=7c09/content-permission-form.pdf) and the following text:

“I request permission for the open-access journal PLOS ONE to publish XXX under the Creative Commons Attribution License (CCAL) CC BY 4.0 (http://creativecommons.org/licenses/by/4.0/). Please be aware that this license allows unrestricted use and distribution, even commercially, by third parties. Please reply and provide explicit written permission to publish XXX under a CC BY license and complete the attached form.”

Please upload the completed Content Permission Form or other proof of granted permissions as an "Other" file with your submission.

In the figure caption of the copyrighted figure, please include the following text: “Reprinted from [ref] under a CC BY license, with permission from [name of publisher], original copyright [original copyright year].”

b. If you are unable to obtain permission from the original copyright holder to publish these figures under the CC BY 4.0 license or if the copyright holder’s requirements are incompatible with the CC BY 4.0 license, please either i) remove the figure or ii) supply a replacement figure that complies with the CC BY 4.0 license. Please check copyright information on all replacement figures and update the figure caption with source information. If applicable, please specify in the figure caption text when a figure is similar but not identical to the original image and is therefore for illustrative purposes only.

The following resources for replacing copyrighted map figures may be helpful:

USGS National Map Viewer (public domain): http://viewer.nationalmap.gov/viewer/

The Gateway to Astronaut Photography of Earth (public domain): http://eol.jsc.nasa.gov/sseop/clickmap/

Maps at the CIA (public domain): https://www.cia.gov/library/publications/the-world-factbook/index.html and https://www.cia.gov/library/publications/cia-maps-publications/index.html

NASA Earth Observatory (public domain): http://earthobservatory.nasa.gov/

Landsat: http://landsat.visibleearth.nasa.gov/

USGS EROS (Earth Resources Observatory and Science (EROS) Center) (public domain): http://eros.usgs.gov/#

Natural Earth (public domain): http://www.naturalearthdata.com/

9. If the reviewer comments include a recommendation to cite specific previously published works, please review and evaluate these publications to determine whether they are relevant and should be cited. There is no requirement to cite these works unless the editor has indicated otherwise.

10. Please review your reference list to ensure that it is complete and correct. If you have cited papers that have been retracted, please include the rationale for doing so in the manuscript text, or remove these references and replace them with relevant current references. Any changes to the reference list should be mentioned in the rebuttal letter that accompanies your revised manuscript. If you need to cite a retracted article, indicate the article’s retracted status in the References list and also include a citation and full reference for the retraction notice.

[Note: HTML markup is below. Please do not edit.]

Reviewers' comments:

Reviewer's Responses to Questions

**Comments to the Author**

1. Is the manuscript technically sound, and do the data support the conclusions?

The manuscript must describe a technically sound piece of scientific research with data that supports the conclusions. Experiments must have been conducted rigorously, with appropriate controls, replication, and sample sizes. The conclusions must be drawn appropriately based on the data presented.

Reviewer #1: Yes

Reviewer #2: Yes

**********

2. Has the statistical analysis been performed appropriately and rigorously?

Reviewer #1: N/A

Reviewer #2: N/A

**********

3. Have the authors made all data underlying the findings in their manuscript fully available?

The PLOS Data policy requires authors to make all data underlying the findings described in their manuscript fully available without restriction, with rare exception (please refer to the Data Availability Statement in the manuscript PDF file). The data should be provided as part of the manuscript or its supporting information, or deposited to a public repository. For example, in addition to summary statistics, the data points behind means, medians and variance measures should be available. If there are restrictions on publicly sharing data—e.g. participant privacy or use of data from a third party—those must be specified.

Reviewer #1: Yes

Reviewer #2: Yes

**********

4. Is the manuscript presented in an intelligible fashion and written in standard English?

PLOS ONE does not copyedit accepted manuscripts, so the language in submitted articles must be clear, correct, and unambiguous. Any typographical or grammatical errors should be corrected at revision, so please note any specific errors here.

Reviewer #1: Yes

Reviewer #2: Yes

**********

5. Review Comments to the Author

Please use the space provided to explain your answers to the questions above. You may also include additional comments for the author, including concerns about dual publication, research ethics, or publication ethics. (Please upload your review as an attachment if it exceeds 20,000 characters)

Reviewer #1: General Introduction comment: I think the introduction could be tightened up a bit. Right now, there’s quite a lot of detail on the global distribution of B. canis, which could be shortened. What feels more central here is Italy’s unique regulatory context and why that made a no-kill strategy necessary. Section 2.2 (“Implementation of the No-Kill Strategy”) would actually fit better in the introduction, since it sets the stage for why this protocol was needed in the first place.

2.5 Post-Adoption Monitoring and Owner Responsibilities: It would be useful to clarify how compliance with follow-up testing was verified (e.g., submission of veterinary reports)

2.4 Seronegative animals were selected for potential rehoming: The quarantine and sequential testing scheme is clearly described, but the rationale for choosing the specific timepoints (days 10, 28, and 56/8 weeks) should be explained. It would also strengthen the section to indicate how many animals tested positive at each stage and to briefly describe housing and welfare conditions during quarantine.

Results section: In the first paragraph of the Results, you report how many dogs tested positive or negative. I think it would give readers a clearer picture if you also included some basic demographics for those groups, like sex ratios or age distribution.

4.5 Study Limitations: The limitations section is strong, but it would be helpful to expand on how the ~20% loss to follow-up could have biased the results, since dogs lost to monitoring may have differed from the ones that followed up. It would also be helpful to explicitly discussing the impact of imperfect test sensitivity and the potential for false negatives would improve the transparency and balance of the interpretation.

Grammar:

1) Brucella canis Outbreak Management (not managment) Towards Rehoming Strategies

2) Introduction first paragraph: (Djokic et al., 2023;) has a stray ; and should be delated and say (Djokic et al., 2023)

3) In the last paragraph of the introduction, there is an et al that does not appear to go with any sentences. Needs to be deleted.

4) Methods: "seronegative status was determined using mSAT, with a cut-off titer of 1:20" (not cu-toff)

5) 96,7% should be a decimal not a comma (96.7%)

Reviewer #2: Overview

The aim of this article is to is to describe the procedure used to manage a Brucella canis outbreak in dogs in Italy. Due to ethical and legislative reasons, the authors chose a no-kill approach with strict serological and bacterial testing of animals prior to selection of consistently negative dogs for rehoming.

According to the authors, of 683 tested dogs, 254 were positive by mSAT and/or bacteriological culture while 438 were negative by both tests. Using the no-kill strategy 398 negative dogs were rehomed.

Major comments

Lines 40 to 51: Insert the references where appropriate for easier identification of which reference supports which statement.

Table 1: As there is a clear difference in management between males and female dogs having the test results by sex would provide more information to the reader.

Minor comments

Throughout the manuscript: Improper use of grammar and spelling mistakes. Please check.

Conclusion

This work has potential for good impact in the scientific community, and we recommend this paper with minor revisions.

**********

6. PLOS authors have the option to publish the peer review history of their article (what does this mean?). If published, this will include your full peer review and any attached files.

If you choose “no”, your identity will remain anonymous but your review may still be made public.

**Do you want your identity to be public for this peer review?**  For information about this choice, including consent withdrawal, please see our Privacy Policy.

Reviewer #1: No

Reviewer #2: No

**********

[NOTE: If reviewer comments were submitted as an attachment file, they will be attached to this email and accessible via the submission site. Please log into your account, locate the manuscript record, and check for the action link "View Attachments". If this link does not appear, there are no attachment files.]

While revising your submission, please upload your figure files to the Preflight Analysis and Conversion Engine (PACE) digital diagnostic tool, https://pacev2.apexcovantage.com/. PACE helps ensure that figures meet PLOS requirements. To use PACE, you must first register as a user. Registration is free. Then, login and navigate to the UPLOAD tab, where you will find detailed instructions on how to use the tool. If you encounter any issues or have any questions when using PACE, please email PLOS at figures@plos.org. Please note that Supporting Information files do not need this step.

# Author comment: Author response to Decision Letter 1

**DOI:** 10.1371/journal.pone.0351742.r002

7 Apr 2026

Dear Reviewers,

We would like to sincerely thank you for your careful review and constructive feedback on our manuscript. Your comments have helped us improve the clarity, accuracy, and overall quality of our work. Please find our detailed point-by-point responses below (and also in "Response to Reviewers" file).

JOURNAL REQUIREMENTS

Comment:

When submitting your revision, we need you to address these additional requirements.

1. Please ensure that your manuscript meets PLOS ONE's style requirements, including those for file naming. The PLOS ONE style templates can be found at https://journals.plos.org/plosone/s/file?id=wjVg/PLOSOne_formatting_sample_main_body.pdf and https://journals.plos.org/plosone/s/file?id=ba62/PLOSOne_formatting_sample_title_authors_affiliations.pdf

Response:

Thank you for this comment. We have readapted the entire manuscript following PLOS ONE’s style requirements.

Comment:

2. We noticed you have some minor occurrence of overlapping text with the following previous publication(s), which needs to be addressed:

- doi: 10.12834/VetIt.2561.16874.1

In your revision ensure you cite all your sources (including your own works), and quote or rephrase any duplicated text outside the methods section. Further consideration is dependent on these concerns being addressed.

Response:

Thank you for this observation. We corrected the citation.

Comment:

3. Your ethics statement should only appear in the Methods section of your manuscript. If your ethics statement is written in any section besides the Methods, please move it to the Methods section and delete it from any other section. Please ensure that your ethics statement is included in your manuscript, as the ethics statement entered into the online submission form will not be published alongside your manuscript.

Response:

Thank you. We moved the ethics statement at the end of the methods section.

Comment:

4. We note that your Data Availability Statement is currently as follows: All relevant data are within the manuscript and its Supporting Information files.

Please confirm at this time whether or not your submission contains all raw data required to replicate the results of your study. Authors must share the “minimal data set” for their submission. PLOS defines the minimal data set to consist of the data required to replicate all study findings reported in the article, as well as related metadata and methods (https://journals.plos.org/plosone/s/data-availability#loc-minimal-data-set-definition).

For example, authors should submit the following data:

- The values behind the means, standard deviations and other measures reported;

- The values used to build graphs;

- The points extracted from images for analysis.

Authors do not need to submit their entire data set if only a portion of the data was used in the reported study.

If your submission does not contain these data, please either upload them as Supporting Information files or deposit them to a stable, public repository and provide us with the relevant URLs, DOIs, or accession numbers. For a list of recommended repositories, please see https://journals.plos.org/plosone/s/recommended-repositories.

If there are ethical or legal restrictions on sharing a de-identified data set, please explain them in detail (e.g., data contain potentially sensitive information, data are owned by a third-party organization, etc.) and who has imposed them (e.g., an ethics committee). Please also provide contact information for a data access committee, ethics committee, or other institutional body to which data requests may be sent. If data are owned by a third party, please indicate how others may request data access.

Response:

Thank you for this comment. Following legal restrictions, data set cannot be disclosed but only in an aggregate way.

Comment:

5. Please note that funding information should not appear in any section or other areas of your manuscript. We will only publish funding information present in the Funding Statement section of the online submission form. Please remove any funding-related text from the manuscript.

Response:

Thank you for the observation. We deleted the funding information from the manuscript.

Comment:

6. Please amend either the title on the online submission form (via Edit Submission) or the title in the manuscript so that they are identical.

Response:

Thank you. We changed the file naming and now they are identical.

Comment:

7. Please ensure that you refer to Figure 1, 2, and 3 in your text as, if accepted, production will need this reference to link the reader to the figure.

Response:

Thank you. We refer to figure 1,2 and 3 in the text, with the following descriptions:

Figure 1: negative and positive dogs work flow; Figure 2: Number of dogs rehomed per municipality; Figure 3: Number of dogs tested per municipality.

Comment:

8. We note that Figure(s) 2 and 3 in your submission contain [map/satellite] images which may be copyrighted. All PLOS content is published under the Creative Commons Attribution License (CC BY 4.0), which means that the manuscript, images, and Supporting Information files will be freely available online, and any third party is permitted to access, download, copy, distribute, and use these materials in any way, even commercially, with proper attribution. For these reasons, we cannot publish previously copyrighted maps or satellite images created using proprietary data, such as Google software (Google Maps, Street View, and Earth). For more information, see our copyright guidelines: http://journals.plos.org/plosone/s/licenses-and-copyright.

We require you to either (1) present written permission from the copyright holder to publish these figures specifically under the CC BY 4.0 license, or (2) remove the figures from your submission:

a. You may seek permission from the original copyright holder of Figure(s) 2 and 3 to publish the content specifically under the CC BY 4.0 license.

We recommend that you contact the original copyright holder with the Content Permission Form (http://journals.plos.org/plosone/s/file?id=7c09/content-permission-form.pdf) and the following text:

“I request permission for the open-access journal PLOS ONE to publish XXX under the Creative Commons Attribution License (CCAL) CC BY 4.0 (http://creativecommons.org/licenses/by/4.0/). Please be aware that this license allows unrestricted use and distribution, even commercially, by third parties. Please reply and provide explicit written permission to publish XXX under a CC BY license and complete the attached form.”

Please upload the completed Content Permission Form or other proof of granted permissions as an "Other" file with your submission.

In the figure caption of the copyrighted figure, please include the following text: “Reprinted from [ref] under a CC BY license, with permission from [name of publisher], original copyright [original copyright year].”

b. If you are unable to obtain permission from the original copyright holder to publish these figures under the CC BY 4.0 license or if the copyright holder’s requirements are incompatible with the CC BY 4.0 license, please either i) remove the figure or ii) supply a replacement figure that complies with the CC BY 4.0 license. Please check copyright information on all replacement figures and update the figure caption with source information. If applicable, please specify in the figure caption text when a figure is similar but not identical to the original image and is therefore for illustrative purposes only.

The following resources for replacing copyrighted map figures may be helpful:

USGS National Map Viewer (public domain): http://viewer.nationalmap.gov/viewer/

The Gateway to Astronaut Photography of Earth (public domain): http://eol.jsc.nasa.gov/sseop/clickmap/

Maps at the CIA (public domain): https://www.cia.gov/library/publications/the-world-factbook/index.html and https://www.cia.gov/library/publications/cia-maps-publications/index.html

NASA Earth Observatory (public domain): http://earthobservatory.nasa.gov/

Landsat: http://landsat.visibleearth.nasa.gov/

USGS EROS (Earth Resources Observatory and Science (EROS) Center) (public domain): http://eros.usgs.gov/#

Natural Earth (public domain): http://www.naturalearthdata.com/

Response:

Thank you for the observation. For figure 2 and 3 we can mention this statement: geographical data © ISTAT – Licence CC-BY 4.0 – www.istat.it

Comment:

9. If the reviewer comments include a recommendation to cite specific previously published works, please review and evaluate these publications to determine whether they are relevant and should be cited. There is no requirement to cite these works unless the editor has indicated otherwise.

Response:

Thank you. We reviewed the reference list.

Comment:

10. Please review your reference list to ensure that it is complete and correct. If you have cited papers that have been retracted, please include the rationale for doing so in the manuscript text, or remove these references and replace them with relevant current references. Any changes to the reference list should be mentioned in the rebuttal letter that accompanies your revised manuscript. If you need to cite a retracted article, indicate the article’s retracted status in the References list and also include a citation and full reference for the retraction notice.

Response:

Thank you for this comment. We rearranged the reference list following the PLOS ONE’s requirements.

REVIEWER’S COMMENT

Reviewer 1

Comment:

General Introduction comment: I think the introduction could be tightened up a bit. Right now, there’s quite a lot of detail on the global distribution of B. canis, which could be shortened. What feels more central here is Italy’s unique regulatory context and why that made a no-kill strategy necessary. Section 2.2 (“Implementation of the No-Kill Strategy”) would actually fit better in the introduction, since it sets the stage for why this protocol was needed in the first place.

Response:

Thank you for the suggestion. We rearranged and tightened the introduction section.

Comment:

2.5 Post-Adoption Monitoring and Owner Responsibilities: It would be useful to clarify how compliance with follow-up testing was verified (e.g., submission of veterinary reports)

Response:

Thank you for this comment. Compliance was verified by CNR testing. There was no provision for sending periodic veterinary reports, because the adoptions were made in large areas, under the management of different veterinary services with distinct competences. This was addressed in the conclusions.

Comment:

2.4 Seronegative animals were selected for potential rehoming: The quarantine and sequential testing scheme is clearly described, but the rationale for choosing the specific timepoints (days 10, 28, and 56/8 weeks) should be explained. It would also strengthen the section to indicate how many animals tested positive at each stage and to briefly describe housing and welfare conditions during quarantine.

Response:

We appreciate the reviewer’s insightful comments. We have now expanded this section to clarify the rationale behind the chosen quarantine timepoints and to provide additional details on testing outcomes and animal welfare during quarantine.

The testing intervals (days 10, 28, and week 8) were selected based on (i) the known kinetics of B. canis infection, including the typical window for seroconversion; (ii) the intermittent nature of bacteraemia and urinary shedding; and (iii) the need to balance diagnostic accuracy with feasible management of a large canine population. The day 10 assessment allowed early detection of dogs that might have been incubating the infection at entry; the day 28 testing corresponded to the expected timeframe for seroconversion in animals exposed shortly before the start of quarantine; and the final evaluation at 8 weeks provided a sufficient temporal buffer to identify delayed serological or bacteriological positives before rehoming. These intervals were therefore chosen to minimize the risk of releasing dogs with early, subclinical, or intermittently detectable infections.

Second, we have added information indicating how many seronegative dogs converted to positive status at each quarantine stage. A small proportion of dogs tested positive during the first and second testing rounds and were consequently removed from the rehoming pathway and returned to the outbreak facility for continued management. No dogs that remained negative through all testing rounds converted at later timepoints. We have clarified these circumstances in the revised text.

Finally, we have expanded the description of housing and welfare conditions during quarantine. Dogs were kept in a dedicated, physically separated facility under strict biosecurity measures. Housing adhered to national animal-welfare standards, ensuring adequate space, environmental enrichment, climate control, and daily monitoring by veterinary staff. Additionally, caretakers used individualized equipment and PPE to reduce cross-contamination risks. These details are now included to provide assurance that the quarantine procedures safeguarded both animal well-being and biosafety.

Comment:

Results section: In the first paragraph of the Results, you report how many dogs tested positive or negative. I think it would give readers a clearer picture if you also included some basic demographics for those groups, like sex ratios or age distribution.

Response:

Thank you for this helpful suggestion. We agree that providing basic demographic data would give the reader a clearer understanding of the population structure and may help contextualize the testing results (De Massis et al., 3032).

We have therefore expanded the first paragraph of the Results section to include sex distribution and age ranges for both the positive and negative groups, reporting the data present in De Massis et al., Including these demographics enables readers to better appreciate whether infection patterns were associated with sex or age, and it supports more transparent interpretation of the outbreak profile.

Comments:

4.5 Study Limitations: The limitations section is strong, but it would be helpful to expand on how the ~20% loss to follow-up could have biased the results, since dogs lost to monitoring may have differed from the ones that followed up. It would also be helpful to explicitly discussing the impact of imperfect test sensitivity and the potential for false negatives would improve the transparency and balance of the interpretation.

Response:

Thank you for this valuable suggestion. We have expanded the Limitations section to more explicitly address the potential biases associated with the ~20% loss to follow-up and to discuss more clearly how imperfect test sensitivity may influence interpretation of the results.

Specifically, we now clarify that dogs lost to follow-up may not be representative of those who completed all scheduled post-adoption tests. These animals could differ in owner compliance, access to veterinary care, or underlying health conditions, introducing the possibility that undetected late infections occurred outside the monitoring framework. Although all rehomed dogs underwent a rigorous multi-step testing protocol before adoption, this attrition still represents a source of uncertainty that may modestly affect the estimated reliability of the rehoming strategy.

Additionally, we have strengthened the discussion of diagnostic test performance, explicitly acknowledging that the mSAT and bacteriological culture—despite their high but imperfect sensitivity—carry an inherent risk of false-negative outcomes. Because B. canis is characterized by intermittent shedding and variable antibody responses, negative results, even when repeated, cannot absolutely exclude infection. We highlight that this limitation necessitates cautious interpretation of our findings and underscores the importance of continued long-term monitoring and the development of more sensitive diagnostic methods.

These revisions enhance transparency and provide a more balanced assessment of the study’s conclusions.

Comment:

Grammar:

1) Brucella canis Outbreak Management (not managment) Towards Rehoming Strate

Attachment Submitted filename: Reply to the Editor on Data disclosing.docx

# Peer review: Decision Letter 1

**DOI:** 10.1371/journal.pone.0351742.r003

**Authors:** Roy Roop, Roy Roop

24 Apr 2026

PONE-D-25-43850R1

*Brucella canis*  Outbreak Management Towards Rehoming Strategies: The Italian Experience

PLOS One

Dear Dr. D'Aurelio,

Thank you for submitting your manuscript to PLOS ONE. After careful consideration, we feel that it has merit but does not fully meet PLOS ONE’s publication criteria as it currently stands. Therefore, we invite you to submit a revised version of the manuscript that addresses the points raised during the review process.

Both reviewers expressed the opinion that your manuscript was greatly improved and that you had addressed the specific concerns raised in their previous reviews. Both, however, have made suggestions for minor grammatical and typographical revisions aimed at improving the 'polish' of  the final version of  the paper. Considering that PLOS ONE leaves this last step to the authors and not to professional editors, I am going to ask that you submit a revised version of  the manuscript addressing the reviewers' suggestions. Given that these are minor changes they should not take long to address.

Please submit your revised manuscript by Jun 08 2026 11:59PM. If you will need more time than this to complete your revisions, please reply to this message or contact the journal office at plosone@plos.org. When you're ready to submit your revision, log on to https://www.editorialmanager.com/pone/ and select the 'Submissions Needing Revision' folder to locate your manuscript file.

Please include the following items when submitting your revised manuscript:

- A letter that responds to each point raised by the academic editor and reviewer(s). You should upload this letter as a separate file labeled 'Response to Reviewers'.
- A marked-up copy of your manuscript that highlights changes made to the original version. You should upload this as a separate file labeled 'Revised Manuscript with Track Changes'.
- An unmarked version of your revised paper without tracked changes. You should upload this as a separate file labeled 'Manuscript'.

If you would like to make changes to your financial disclosure, please include your updated statement in your cover letter. Guidelines for resubmitting your figure files are available below the reviewer comments at the end of this letter.

If applicable, we recommend that you deposit your laboratory protocols in protocols.io to enhance the reproducibility of your results. Protocols.io assigns your protocol its own identifier (DOI) so that it can be cited independently in the future. For instructions see: https://journals.plos.org/plosone/s/submission-guidelines#loc-laboratory-protocols. Additionally, PLOS ONE offers an option for publishing peer-reviewed Lab Protocol articles, which describe protocols hosted on protocols.io. Read more information on sharing protocols at https://plos.org/protocols?utm_medium=editorial-email&utm_source=authorletters&utm_campaign=protocols.

As the corresponding author, your ORCID iD is verified in the submission system and will appear in the published article. PLOS supports the use of ORCID, and we encourage all coauthors to register for an ORCID iD and use it as well. Please encourage your coauthors to verify their ORCID iD within the submission system before final acceptance, as unverified ORCID iDs will not appear in the published article. *Only*  the individual author can complete the verification step; PLOS staff *cannot*  verify ORCID iDs on behalf of authors.

We look forward to receiving your revised manuscript!

Sincerely,

Marty Roop, Ph.D.

Academic Editor

*PLOS One*

Journal Requirements:

If the reviewer comments include a recommendation to cite specific previously published works, please review and evaluate these publications to determine whether they are relevant and should be cited. There is no requirement to cite these works unless the editor has indicated otherwise.

Please review your reference list to ensure that it is complete and correct. If you have cited papers that have been retracted, please include the rationale for doing so in the manuscript text, or remove these references and replace them with relevant current references. Any changes to the reference list should be mentioned in the rebuttal letter that accompanies your revised manuscript. If you need to cite a retracted article, indicate the article’s retracted status in the References list and also include a citation and full reference for the retraction notice.

[Note: HTML markup is below. Please do not edit.]

Reviewers' comments:

Reviewer's Responses to Questions

**Comments to the Author**

1. If the authors have adequately addressed your comments raised in a previous round of review and you feel that this manuscript is now acceptable for publication, you may indicate that here to bypass the “Comments to the Author” section, enter your conflict of interest statement in the “Confidential to Editor” section, and submit your "Accept" recommendation.

Reviewer #1: All comments have been addressed

Reviewer #2: All comments have been addressed

**********

2. Is the manuscript technically sound, and do the data support the conclusions?

The manuscript must describe a technically sound piece of scientific research with data that supports the conclusions. Experiments must have been conducted rigorously, with appropriate controls, replication, and sample sizes. The conclusions must be drawn appropriately based on the data presented.

Reviewer #1: Yes

Reviewer #2: Yes

**********

3. Has the statistical analysis been performed appropriately and rigorously?

Reviewer #1: Yes

Reviewer #2: Yes

**********

4. Have the authors made all data underlying the findings in their manuscript fully available?

The PLOS Data policy requires authors to make all data underlying the findings described in their manuscript fully available without restriction, with rare exception (please refer to the Data Availability Statement in the manuscript PDF file). The data should be provided as part of the manuscript or its supporting information, or deposited to a public repository. For example, in addition to summary statistics, the data points behind means, medians and variance measures should be available. If there are restrictions on publicly sharing data—e.g. participant privacy or use of data from a third party—those must be specified.

Reviewer #1: Yes

Reviewer #2: Yes

**********

5. Is the manuscript presented in an intelligible fashion and written in standard English?

PLOS ONE does not copyedit accepted manuscripts, so the language in submitted articles must be clear, correct, and unambiguous. Any typographical or grammatical errors should be corrected at revision, so please note any specific errors here.

Reviewer #1: No

Reviewer #2: Yes

**********

6. Review Comments to the Author

Please use the space provided to explain your answers to the questions above. You may also include additional comments for the author, including concerns about dual publication, research ethics, or publication ethics. (Please upload your review as an attachment if it exceeds 20,000 characters)

Reviewer #1: The authors have addressed the reviewer comments thoroughly and substantially improved the manuscript, particularly in the Methods, Results, and Limitations sections. The study is well-structured and provides valuable insight into B. canis outbreak management. A few minor grammatical issues still remain. Pending these minor revisions, I recommend acceptance.

1) "discospondlytis”: should be “discospondylitis

2) Bracket error: “Africa 12], and Europe [1]”

3) Miss space after "programs: "absence of mandatory surveillance programs[1]"

4) This sentence appears twice so need to delete one instance: “Because B. canis is characterized by intermittent shedding…”

5) Broken/fragmented sentence and not sure where the et al was suppose to go to: "In 2020, B. canis has been detected and isolated for the first time in a commercial breeding kennel [18]. et al."

Reviewer #2: Overview

Thank you for your careful revision and detailed response. The manuscript is substantially improved in clarity and methodological transparency. Most of my prior concerns have been addressed; just a few edits are required.

Point-by-point assessment relative to my previous review:

Major comments

Lines 40 to 51: Insert the references where appropriate for easier identification of which reference supports which statement.

- Assessment: The authors included references for each statement.

Table 1: As there is a clear difference in management between males and female dogs having the test results by sex would provide more information to the reader.

- Assessment: The authors have included additional tables which provide a breakdown of the serological and culture results per sex for better understanding.

Minor comments

Line 59: It seems that the sentence starts with a “et al.” which does not seem connected to any sentence. It needs to be removed.

Table 1: The first line of the table reads “Serology (Msat)” where it should read “Serology (mSAT)”. Please make the appropriate change.

**********

7. PLOS authors have the option to publish the peer review history of their article (what does this mean?). If published, this will include your full peer review and any attached files.

If you choose “no”, your identity will remain anonymous but your review may still be made public.

**Do you want your identity to be public for this peer review?**  For information about this choice, including consent withdrawal, please see our Privacy Policy.

Reviewer #1: No

Reviewer #2: No

**********

[NOTE: If reviewer comments were submitted as an attachment file, they will be attached to this email and accessible via the submission site. Please log into your account, locate the manuscript record, and check for the action link "View Attachments". If this link does not appear, there are no attachment files.]

To ensure your figures meet our technical requirements, please review our figure guidelines: https://journals.plos.org/plosone/s/figures

You may also use PLOS’s free figure tool, NAAS, to help you prepare publication quality figures: https://journals.plos.org/plosone/s/figures#loc-tools-for-figure-preparation.

NAAS will assess whether your figures meet our technical requirements by comparing each figure against our figure specifications.

# Author comment: Author response to Decision Letter 2

**DOI:** 10.1371/journal.pone.0351742.r004

28 May 2026

Dear Reviewers,

We would like to sincerely thank you for your careful review and constructive feedback on our manuscript. Your comments have helped us improve the clarity, accuracy, and overall quality of our work. Please find our detailed point-by-point responses below.

Reviewer #1

The authors have addressed the reviewer comments thoroughly and substantially improved the manuscript, particularly in the Methods, Results, and Limitations sections. The study is well-structured and provides valuable insight into B. canis outbreak management. A few minor grammatical issues still remain. Pending these minor revisions, I recommend acceptance.

Comment: 1) "discospondlytis”: should be “discospondylitis

Response: Thank you for this comment, we corrected the term.

Comment: 2) Bracket error: “Africa 12], and Europe [1]”

Response: Thank you, we added the bracket.

Comment: 3) Miss space after "programs: "absence of mandatory surveillance programs[1]"

Response: Thank you for this observation, we added the space after “programs”.

Comment: 4) This sentence appears twice so need to delete one instance: “Because B. canis is characterized by intermittent shedding…”

Response: Thank you for the comment, we deleted the second one.

Comment: 5) Broken/fragmented sentence and not sure where the et al was suppose to go to: "In 2020, B. canis has been detected and isolated for the first time in a commercial breeding kennel [18]. et al."

Response: Thank you for the observation, that “et al.” was a typo, we deleted it.

Reviewer #2

Thank you for your careful revision and detailed response. The manuscript is substantially improved in clarity and methodological transparency. Most of my prior concerns have been addressed; just a few edits are required.

Point-by-point assessment relative to my previous review:

Major comments

Lines 40 to 51: Insert the references where appropriate for easier identification of which reference supports which statement.

- Assessment: The authors included references for each statement.

Table 1: As there is a clear difference in management between males and female dogs having the test results by sex would provide more information to the reader.

- Assessment: The authors have included additional tables which provide a breakdown of the serological and culture results per sex for better understanding.

Minor comments

Comment: Line 59: It seems that the sentence starts with a “et al.” which does not seem connected to any sentence. It needs to be removed.

Response: Thank you for this observation. As written previously, that “et al.” was a typo, we deleted it.

Comment: Table 1: The first line of the table reads “Serology (Msat)” where it should read “Serology (mSAT)”. Please make the appropriate change. Review Comments to the Author

Response: Thank you for the comment, we changed it.

Attachment Submitted filename: Rebuttal letter_05.docx

# Editor report: Decision Letter 2

**DOI:** 10.1371/journal.pone.0351742.r005

**Authors:** Roy Roop, Roy Roop, Roy Roop

1 Jun 2026

*Brucella canis*  Outbreak Management Towards Rehoming Strategies: The Italian Experience

PONE-D-25-43850R2

Dear Dr. D'Aurelio,

We’re pleased to inform you that your manuscript has been judged scientifically suitable for publication and will be formally accepted for publication once it meets all outstanding technical requirements.

Within one week, you’ll receive an e-mail detailing the required amendments. When these have been addressed, you’ll receive a formal acceptance letter and your manuscript will be scheduled for publication.

An invoice will be generated when your article is formally accepted. Please note, if your institution has a publishing partnership with PLOS and your article meets the relevant criteria, all or part of your publication costs will be covered. Please make sure your user information is up-to-date by logging into Editorial Manager at Editorial Manager® and clicking the ‘Update My Information' link at the top of the page. For questions related to billing, please contact billing support.

If your institution or institutions have a press office, please notify them about your upcoming paper to help maximize its impact. If they’ll be preparing press materials, please inform our press team as soon as possible -- no later than 48 hours after receiving the formal acceptance. Your manuscript will remain under strict press embargo until 2 pm Eastern Time on the date of publication. For more information, please contact onepress@plos.org.

Sincerely,

R. Martin Roop II, Ph.D.

Academic Editor

*PLOS One*

Additional Editor Comments (optional):

Reviewers' comments:

# Editor report: Acceptance letter

**DOI:** 10.1371/journal.pone.0351742.r006

**Authors:** Roy Roop, Roy Roop, Roy Roop

PONE-D-25-43850R2

PLOS One

Dear Dr. D'Aurelio,

I'm pleased to inform you that your manuscript has been deemed suitable for publication in PLOS One. Congratulations! Your manuscript is now being handed over to our production team.

At this stage, our production department will prepare your paper for publication. This includes ensuring the following:

* All references, tables, and figures are properly cited

* All relevant supporting information is included in the manuscript submission,

* There are no issues that prevent the paper from being properly typeset

You will receive further instructions from the production team, including instructions on how to review your proof when it is ready. Please keep in mind that we are working through a large volume of accepted articles, so please give us a few days to review your paper and let you know the next and final steps.

Lastly, if your institution or institutions have a press office, please let them know about your upcoming paper now to help maximize its impact. If they'll be preparing press materials, please inform our press team within the next 48 hours. Your manuscript will remain under strict press embargo until 2 pm Eastern Time on the date of publication. For more information, please contact onepress@plos.org.

You will receive an invoice from PLOS for your publication fee after your manuscript has reached the completed accept phase. If you receive an email requesting payment before acceptance or for any other service, this may be a phishing scheme. Learn how to identify phishing emails and protect your accounts at https://explore.plos.org/phishing.

If we can help with anything else, please email us at customercare@plos.org.

Thank you for submitting your work to PLOS ONE and supporting open access.

Kind regards,

PLOS ONE Editorial Office Staff

on behalf of

Dr. Roy Martin Roop II

Academic Editor

PLOS One
